# Elucidating the domain architecture and functions of non-core RAG1: The capacity of a non-core zinc-binding domain to function in nuclear import and nucleic acid binding

**DOI:** 10.1186/1471-2091-12-23

**Published:** 2011-05-20

**Authors:** Janeen L Arbuckle, Negar S Rahman, Shuying Zhao, William Rodgers, Karla K Rodgers

**Affiliations:** 1 Department of Biochemistry and Molecular Biology, The University of Oklahoma Health Sciences Center, Oklahoma City, Oklahoma 73190, USA; 2 Cardiovascular Biology Program, Oklahoma Medical Research Foundation, Oklahoma City, Oklahoma 73104, USA; 3 Department of Obstetrics and Gynecology, University of Alabama, Birmingham, Alabama 35294, USA; 4 Department of Molecular Virology and Microbiology, Baylor College of Medicine, Houston, Texas 77030, USA

## Abstract

**Background:**

The repertoire of the antigen-binding receptors originates from the rearrangement of immunoglobulin and T-cell receptor genetic loci in a process known as V(D)J recombination. The initial site-specific DNA cleavage steps of this process are catalyzed by the lymphoid specific proteins RAG1 and RAG2. The majority of studies on RAG1 and RAG2 have focused on the minimal, core regions required for catalytic activity. Though not absolutely required, non-core regions of RAG1 and RAG2 have been shown to influence the efficiency and fidelity of the recombination reaction.

**Results:**

Using a partial proteolysis approach in combination with bioinformatics analyses, we identified the domain boundaries of a structural domain that is present in the 380-residue N-terminal non-core region of RAG1. We term this domain the Central Non-core Domain (CND; residues 87-217).

**Conclusions:**

We show how the CND alone, and in combination with other regions of non-core RAG1, functions in nuclear localization, zinc coordination, and interactions with nucleic acid. Together, these results demonstrate the multiple roles that the non-core region can play in the function of the full length protein.

## Background

Development of the adaptive immune system relies on the coordinated assembly of the genes encoding immunoglobulin and T cell receptor subunits in a process known as V(D)J recombination [1]. In this process, one of each type of component variable (V), diversity (D) and joining (J) gene segments are combined to form the coding sequence of the antigen binding regions. Due in part to the array of potential gene segment combinations, V(D)J recombination events lead to the vast sequence diversity in the antigen receptor repertoire. The site-specific DNA cleavage reactions in V(D)J recombination are catalyzed by the lymphoid specific proteins RAG1 and RAG2 in a cell lineage and stage specific manner. The recombination signal sequence (RSS) that flanks each gene segment directs the RAG proteins to the appropriate DNA cleavage sites. The RSS consists of both a conserved heptamer and a nonamer sequence separated by a poorly conserved spacer of either 12 or 23 base pairs. Appropriate recombination only occurs between gene segments flanked by RSSs of dissimilar spacer lengths, a requirement referred to as the 12/23 rule.

V(D)J recombination occurs in two distinct phases, the first of which relies largely on the RAG proteins. During the first phase of recombination, the RAG proteins assemble on the RSSs of the two gene segments to be recombined, forming a pre-cleavage synaptic (or paired) complex. The proteins first generate a single-strand nick 5' of the heptamer sequence of the RSS, producing a free 3' hydroxyl group on the coding gene segment. This hydroxyl group subsequently attacks the opposing strand in a direct transesterification reaction, generating a double-strand break at the coding gene:RSS border [2]. Under physiological conditions, hairpin formation requires that the RAG proteins bind to both a 12-RSS and 23-RSS in a paired complex [3-8]. The generation of double-strand breaks is therefore coordinated at the two RSSs undergoing recombination, assuring that double-strand breaks are not made at isolated RSSs. The products of this first phase of recombination are blunt-ended RSSs and covalently sealed coding gene segments. The second phase of recombination involves the opening, processing, and subsequent joining of the covalently sealed hairpin structures and the RSS signal ends to form coding and signal joints, respectively. This phase relies on the action of the ubiquitously expressed proteins of the non-homologous end-joining (NHEJ) DNA repair pathway [9], although the RAG proteins may function in this phase by ensuring proper DNA repair through the NHEJ machinery [4,10-13].

Early studies of the RAG proteins identified the minimal regions of the proteins required for catalysis [14-17]. These regions, referred to as the core proteins, demonstrated improved solubility over their full-length counterparts and have therefore served as the basis for most biochemical studies of the RAG proteins [18]. Murine core RAG1 consists of residues 384-1008 from the 1040 residue full length protein, and murine core RAG2 includes residues 1-387 from the 527 residue full length protein. Core RAG1 consists of multiple structural domains, termed the nonamer binding domain (NBD; residues 389-464) [19], and the central (residues 528-760) and C-terminal (residues 761-980) domains [20]. Besides the ability to recognize the RSS nonamer and heptamer through the NBD [19,21,22] and the central domain [20,23], respectively, core RAG1 contains the essential acidic active site residues [24-26]. Core RAG2 is predicted to adopt a six-bladed propeller structure [27] and functions to enhance sequence-specific interactions of RAG1 to the RSS [28,29], and possibly induce conformational changes in RAG1 to activate DNA cleavage activity [30].

The non-core regions of both RAG proteins, though considered "dispensable" for recombination, are well-conserved, suggesting that these regions have a functional role. Notably, a number of studies have shown that core RAG1 and core RAG2 were significantly less efficient in the recombination of both exogenous plasmid substrates and endogenous genetic loci when compared to their full-length counterparts [8,17,31-36]. The impact of this decreased recombination efficiency was shown in experiments using core RAG1 or core RAG2 knock-in mice. Mice expressing either core RAG protein in place of its full-length counterpart demonstrated impaired B and T cell development [35,36], with a reduction in RSS cleavage and limited specific recombination events at both IgH and TCRβ loci [34-36]. Furthermore, recombination products showed an increased frequency of aberrant signal and coding joints, indicating that the non-core regions of the RAG proteins are critical for appropriate recombination at endogenous genetic loci [37].

Multiple previous studies have shown that non-core RAG2 contains a PHD module [38], which binds to Histone H3 di- or trimethylated on Lys 4 (H3K4me3) [39-41]. The interaction with the methylated Histone H3 is required for efficient recombination on chromatinized substrate [39], and also functions to stimulate the catalytic nicking and hairpin formation steps [42]. In a separate role, phosphorylation of Thr 490 is a prerequisite for ubiquitination and degradation of RAG2 at the G1-S transition of the cell cycle [43].

The role of the N-terminal non-core region of RAG1 has been more elusive. There are three separate lines of study that indicate important roles of the non-core region in the function of the RAG1 protein. First, previous mutagenesis studies revealed regions in non-core RAG1 that significantly impacted recombination of exogenous plasmid substrates [33,44], including a conserved basic region (located between residues 218-224) [32] and conserved cysteine residues within the N-terminal 250 residues [33,45]. Second, although most of the non-core region of RAG1 remains structurally undefined, the crystal structure of a conserved zinc dimerization domain (ZDD) between residues 265-380 has been solved [46,47]. Further, the RING finger within this domain exhibits E3 ubiquitin ligase activity, demonstrating autoubiquitination, as well as ubiquitination of an artificial substrate in in vitro assays [48,49]. The RAG1 RING finger has also been shown to facilitate ubiquitination of the nuclear transport protein karyopherin alpha 1 [50] and Histone H3 [51,52]. How the E3 ligase activity on either of these latter targets affects the V(D)J recombination process has yet to be established. Third, regions N-terminal to the ZDD in full length RAG1 have been shown to mediate protein-protein interactions. For example, full-length RAG1 co-purifies with the Ku70/Ku80 heterodimer under low stringency conditions, and this association requires the presence of non-core RAG1 residues 211-383 [53]. In a separate study, a yeast two-hybrid approach demonstrated that a region of non-core RAG1, encompassed by residues 173-250, mediated interactions with the transcription factor GMEB1 and the splicing factor SF3A2 [54]. Although the full implications of these protein-protein interactions are not yet clear, these studies indicate that non-core RAG1, like non-core RAG2, plays multiple roles in the recombination reaction and its regulation.

To further our understanding of RAG1, we have utilized biochemical, bioinformatic, and biophysical approaches to identify the domain composition of the N-terminal non-core region of the protein. In this context, a domain is defined as an independently folded unit that can retain biological activity even if excised from the full length protein. Since domains can participate in key functions of the intact protein, such as providing binding surfaces for macromolecular interactions, it will be critical to determine the domain architecture of non-core RAG1 to obtain a detailed understanding of its function. Here, we have identified domain boundaries that yielded isolation of a structurally independent domain, termed the central non-core domain (CND), which consists of residues 87-217. We show that this domain interacts strongly with zinc ions, preferentially binds double-stranded DNA, and in conjunction with a neighboring non-core region contributes to nuclear localization of the full length RAG1 protein.

## Results

### Identification of domain boundaries in non-core RAG1

**Figure 1:**
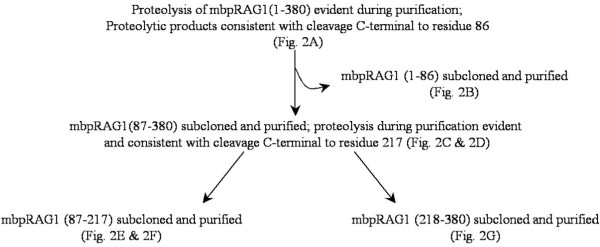
**Summary of steps in the identification of domains in non-core RAG1**.

To characterize the non-core region of RAG1, residues 1-380 of murine RAG1 were expressed as a maltose-binding protein (MBP) fusion construct in *E. coli*. Expression of the non-core region in its entirety resulted in extensive proteolysis and aggregation of the protein. We therefore took a domain approach to characterize the non-core region of RAG1, with the goal that identification and characterization of independent structural domains would provide important insights into the function of non-core RAG1. In this study, we identified a structural domain in the N-terminal non-core region of RAG1, which we term the central non-core domain (CND). Our strategy is outlined in Figure 1.

**Figure 2:**
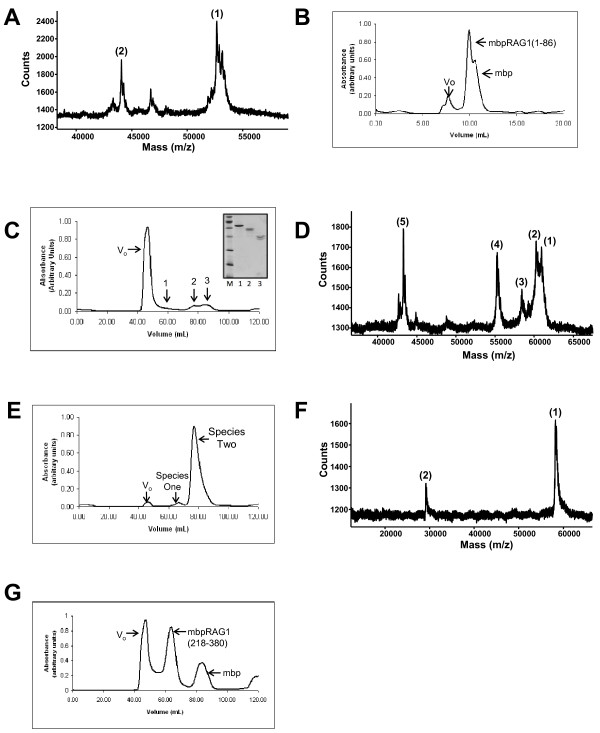
**Identification of domains in non-core RAG1**. **(A) **MALDI-TOF spectrum of proteolytic products generated during purification of MBP-RAG1(1-380). Two peaks were observed with molecular weight (MWt) values: (1) 52,756.4 Da, consistent with cleavage C-terminal to residue 86, and (2) 44,061.9 Da, consistent with cleavage just C-terminal to the MBP portion of the fusion construct. The predicted mass of MBP-RAG1(1-86) is 52,815 Da. **(B) **SEC of MBP-RAG1(1-86). **(C) **SEC of MBP-RAG1(87-380). V_o _denotes the void volume and the position at which aggregated protein elutes. Inset: SEC peaks 1-3 analyzed by SDS-PAGE. (All lanes are from the same gel.) Samples in lanes 1-3 correspond to peaks assigned in the SEC elution profile. **(D) **MALDI-TOF spectrum of peak 2 from the SEC of MBP-RAG1(87-380). Five major peaks were observed with MWt values: (1) 60,902.8, (2) 60,255.1, (3) 58,410.8, (4) 55,239.7, and (5) 43,273 Da. These values are consistent with cleavage C-terminal to residues 238, 232, 217, 190, and the MBP portion of the fusion construct, respectively. Predicted masses for the corresponding MBP-RAG1 polypeptides are 60,897 Da (MBP-RAG1(87-238)), 60,199 Da (MBP-RAG1(87-232)), 58,400 Da (MBP-RAG1(87-217)), and 55,240 Da (MBP-RAG1(87-190)). **(E) **SEC of MBP-CND following subcloning and purification. **(F) **MALDI-TOF spectrum of MBP-CND. The peaks yield MWt values consistent with the +1 (1) and +2 (2) species of the protein. **(G) **SEC of MBP-bZDD following subcloning and purification. All SEC runs were performed using Superdex 200 columns with either 20 ml (panel B) or 200 ml (panels C, E, and G) column volumes.

Noting that proteolysis frequently occurs at domain boundaries [55], we first identified the proteolytic product generated during the expression of MBP-RAG1(1-380). Using MALDI-TOF mass spectrometry, the size of the cleavage product generated during purification was found to be consistent with cleavage of the fusion construct occurring C-terminal to residue 86 (Figure 2A). A fragment consisting of murine RAG1 residues 1-86 was then expressed as an MBP fusion (MBP-RAG1(1-86)). The resultant fusion protein formed a discrete monomeric species during size exclusion chromatography (SEC) (Figure 2B).

Next, we expressed the remaining region of non-core RAG1 as an MBP fusion construct (MBP-RAG1(87-380)) (Figure 2C). Though a small portion of this construct remained full-length throughout purification (peak 1), this protein also underwent extensive proteolysis. Analysis of the primary species observed by SEC (peak 2) by mass spectrometry of MBP-RAG1(87-380) indicated that the molecular weight of the cleavage products was consistent with cleavage occurring C-terminal to residues 190, 217, 232, and 238 (Figure 2D). Subcloning and expression of fusion constructs terminating at either residue 232 or 238 resulted in further proteolysis during purification (not shown). In addition, termination at residue 232 or 238 disrupts the previously described basic regions within non-core RAG1 [31]. Thus, we characterized the fragment consisting of residues 87-217 (the CND). This region contains the conserved cysteine residues previously described [33]. MBP fused to RAG1 residues 87-217 was markedly well behaved, with no detectable proteolysis or aggregation occurring during purification (Figure 2E &2F).

Earlier studies of non-core RAG1 identified and characterized the ZDD, which is located between residues 265-380, and is at the N-terminal border to the core region of the protein [46,47]. A previous study showed that the non-core RAG1 fragment consisting of residues 218-389 catalyzes autoubiquitination at residue 233 [48]. To characterize the biophysical properties of this region, we generated a fusion construct between MBP and residues 218-380 of RAG1 (MBP-RAG1(218-380)). We refer to this fragment as the basic-zinc dimerization domain (bZDD), as it contains both the conserved basic region and the ZDD. Expression of this construct in *E. coli *resulted in fusion protein that eluted from SEC in two peaks, with the peak eluting at V_o _likely containing aggregated protein and the second peak containing more well-behaved protein (Figure 2G).

### Bioinformatic analysis of non-core RAG1

**Figure 3:**
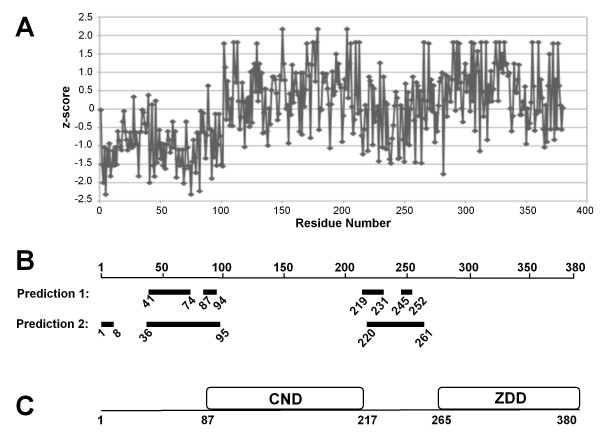
**Bioinformatic analysis of the N-terminal non-core region of RAG1**. **(A) **Sequence conservation of the N-terminal non-core RAG1 region based on phylogenetic analysis. The z-score (y-axis) for each residue is a comparison of the observed conservation at that residue to the mean conservation across all sites in the sequence, and is the number of standard deviations away from the mean value [56]. Positions with a more positive z-score are more highly conserved, whereas positions with a more negative score are less conserved. **(B) **Regions in the N-terminal non-core region of RAG1 predicted to be highly disordered are shown as thick bars. Each bar is labeled with N- and C-terminal residue numbers. Predictions 1 and 2 are results from DripPred and Disopred programs (see Methods), respectively. **(C) **The domain architecture of non-core RAG1 based on the combined experimental results (in Figure 2) and the bioinformatic methods.

Next, we used bioinformatics methods to analyze the non-core region of RAG1 in order to obtain additional corroboration as to the multi-domain architecture of this region of the protein. First, phylogenetic analysis of the N-terminal non-core region was performed using Intrepid [56] and ConSeq [57], with both methods giving similar results. Figure 3A illustrates the results from Intrepid. There is a strikingly higher level of conservation for the region encompassing residues 100-215 as opposed to its flanking sequences. Specifically, the mean z-score (see Figure 3A legend) for residues 100-215 is 0.41, whereas the 50 residues N-terminal and C-terminal to this region have z-scores of -0.3 and -0.2, respectively. The region corresponding to the ZDD is 0.41, also indicating a more highly conserved domain.

Multi-domain proteins can be altered through evolution by the acquisition of domains, which render additional functionalities to the protein [58]. The jawed vertebrate RAG1 proteins have retained the N-terminal non-core region, including that corresponding to the CND, the basic region, and the ZDD. Within the last decade, RAG1-like sequences have also been found in invertebrate species [59-61]. The purple sea urchin RAG1-like sequence contains an extended N-terminal non-core region [60]. The alignment of sea urchin RAG1-like protein and murine RAG1 has been shown in detail in a previous study [60]. Notably, only the region corresponding to residues 100-215 in murine RAG1 is present in the non-core region of the sea urchin RAG1-like sequence. In contrast, the ZDD is replaced with an unrelated sequence consisting of 11 repeats of an 8-residue sequence [60]. Thus, the region corresponding to residues 100-215 likely plays an early role in the evolutionary history of the protein [60,61].

Aside from the zinc binding motifs in the ZDD (RING and ZFA) of non-core RAG1, there is no sequence homology to known protein domains. Difficulties remain in *ab initio *prediction of domain boundaries [62]. However, structural domains are often embedded within disordered or unstructured regions in multi-domain proteins, and methods to predict disordered regions in proteins are proving to be reasonably accurate [63]. Here, we used multiple algorithms to predict disordered regions in the non-core RAG1 sequence, with good agreement between separate methods (Figure 3B). Specifically, the regions predicted to be highly disordered immediately flank either side of residues 100-215. This latter region, which closely corresponds to the CND, and the ZDD are predicted to be highly ordered, consistent with these regions folding into compact structural domains.

It is well acknowledged that the full length RAG1 protein is problematic to work with; and as such it has not been feasible to show that the non-core domains are proteolytically resistant in the mammalian expressed full length protein. However, these bioinformatic results combined with the experimental studies on the bacterially-expressed non-core region (Figure 2) strongly indicate that the domain architecture of the N-terminal non-core region within the full length protein consists of two major structural domains (illustrated in Figure 3C).

It is possible that the first 86 residues of full length RAG1 also fold into an independent domain; however, the low sequence conservation of this region suggests it does not play a central role in RAG1 functions. Moreover, the basic region, which links the CND and ZDD, is predicted to be disordered, consistent with our findings of multiple cleavage sites in this region (Figure 2D).

### Biophysical analysis of the RAG1 CND in self-association, zinc binding, and protein stability

**Figure 4:**
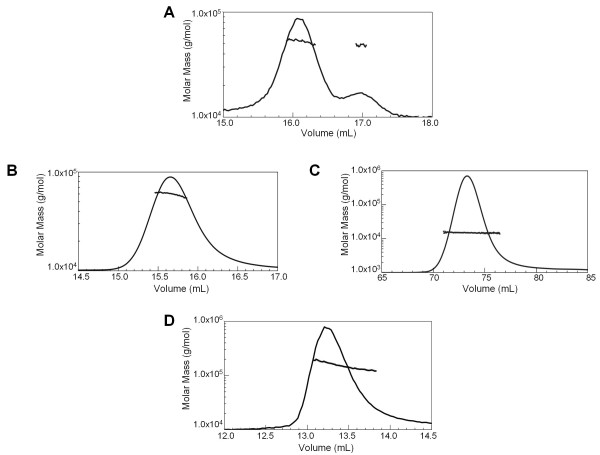
**Self-Association Properties of non-core RAG1 domains**. MALLS-SEC analysis of the non-core RAG1 domains/regions. Molar mass distribution plots are shown for each non-core RAG1 region. The peaks represent the elution profile of the protein sample from a size exclusion column as monitored by a refractometer detector. The molar mass profile measured by light scattering (plotted versus the left axis) of the material that eluted at the corresponding volumes is overlaid on the elution profile. **(A) **The experimentally determined molar mass of MBP-RAG1(1-86) eluted from a 20 mL Superdex 200 column at 52,600 ± 500 Da (predicted MWt for monomeric MBP-RAG1(1-86) is at 52,815 Da). The molar mass for the species with the longer elution time is ~47 ± 1 kDa, which is consistent with that of the MBP portion of the fusion construct alone. **(B) **The predominant form of MBP-CND identified by SEC (see Figure 2E) was pooled, concentrated, and subjected to MALLS-SEC (using a 20 mL Superdex 200 column). The fusion construct eluted as a single species with an observed molar mass of 58,200 ± 700 Da (predicted monomeric MWt is at 58,400 Da). **(C) **Tagless CND (at a loading concentration of 23 μM) eluted from a 120 mL Superdex 75 column as a single species with an observed molar mass of 14,900 ± 300 Da (predicted monomeric MWt is at 15,317 Da). **(D) **MBP-bZDD eluted from a 20 mL Superdex 200 column with an observed molar mass of ~155 kDa (predicted dimeric MWt is at 123,634 Da).

Full length RAG1 likely functions as a dimer, or higher oligomer [64]. Both the core region and the ZDD dimerize when expressed separately and when included in the same fragment (residues 263-1008) [65]. To determine if other regions in the N-terminal non-core region of RAG1 besides the ZDD contributes to dimer formation, SEC coupled with multi-angled laser light scattering (MALLS-SEC) was performed. MALLS-SEC analysis of MBP-RAG1(1-86) and the predominant MBP-CND species (Species 2 in Figure 2E) show that both fusion proteins are monomeric (Figure 4A &4B). The minor form of MBP-CND (Species 1 in Figure 2E) does not appear to represent a functional form of the domain, as the aggregate did not dissociate upon reapplication to SEC (not shown), and as the concentrated monomeric MBP-CND did not form the aggregated species (Figure 4B). MALLS-SEC analysis of a tagless form of the CND (see Methods) confirmed that this domain does not self associate, and that MBP did not interfere with CND oligomerization in the fusion protein (Figure 4C). As expected, MBP-bZDD eluted as a predominantly dimeric species, although with some polydispersity (Figure 4D). The polydispersity of this fragment is likely due to the basic region, as MBP-ZDD was previously shown to be a monodisperse dimer [46]. Based on these results, we conclude that the ZDD is the only domain in non-core RAG1 that can self-associate and contribute to oligomerization of the full-length protein.

The CND contains multiple conserved cysteine and histidine residues, and it was previously suggested that this region of non-core RAG1 may coordinate zinc ions [33]. However, the spacing of these conserved residues is not consistent with any previously described zinc binding motif. To determine if the CND participates in zinc binding, inductively coupled plasma mass spectrometry (ICP-MS) and flame atomic absorption spectroscopy (FAAS) methods were used. The two methods show that the CND (both the MBP fusion protein and the tagless form) binds two zinc ions (Table 1). As a control the zinc content of MBP-bZDD was also determined, and measured at four zinc ions per MBP-bZDD monomer, consistent with the known zinc-binding stoichiometry for the ZDD (Table 1) [47]. Treatment of CND with the chelator DTPA was sufficient to remove the majority of zinc from the protein (Table 1). Subsequently, the zinc-free form of CND, referred to as apoCND, failed to re-bind zinc ions, indicating that the removal of zinc from CND may cause an irreversible structural change in the domain under the conditions used here. Potential zinc-coordinated residues were mutated in three separate mutants, C110,113A, C175,178A, and C210,213A, which were expressed and purified as MBP fusions from *E. coli *BL21 cells. Although the mutants were expressed in lon-deficient *E. coli *BL21 cells, each CND mutant underwent significant proteolysis during purification, consistent with the importance of these residues in the structural integrity of the CND (not shown).

**Table 1:** Zinc:Protein Ratio for CND and bZDD

RAG1 Domain	Conditions	**Zinc:Protein Ratio ICP-MS**^**a,b**^	**Zinc:Protein Ratio FAAS**^**a,b**^
CND^c^	Zinc Free Buffer	2.08 ± 0.68	2.00 ± 0.44
CND^c^	DTPA Buffer	0.24 ± 0.14	0.37 ± 0.29
bZDD^d^	Zinc Free Buffer	4.23 ± 0.65	4.12 ± 0.78

a The zinc content of each construct was determined using ICP-MS and FAAS.

b Data presented represent the average of two or more independent experiments.

c Zinc binding results for mbp-CND and tagless CND were comparable. The results presented for CND represent the average from both data sets.

d Zinc binding for bZDD was evaluated for the mbp-tagged construct only.

**Figure 5:**
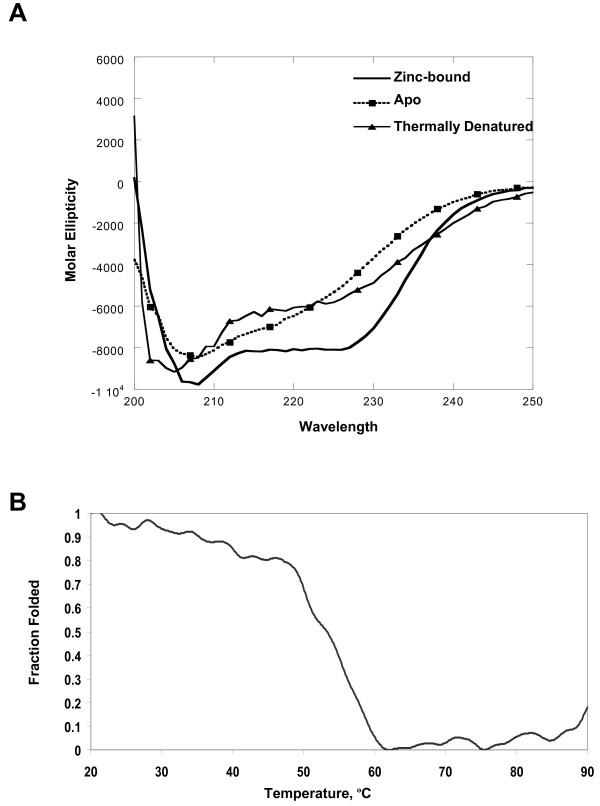
**Secondary structure analysis of CND using CD spectroscopy**. **(A) **CD spectra of tagless CND under various conditions including zinc-bound, after treatment with a chelating agent (apo) and after slowly heating the protein to 90°C (thermally denatured). **(B) **Thermal denaturation of tagless CND as determined by changes in signal intensity at a wavelength of 222 nm. Fraction folded was calculated as described in Methods.

To characterize the secondary structure of the CND, the MBP portion of the fusion construct was removed and tagless CND purified. Investigation of CND by far UV circular dichroism (CD) spectroscopy (at 20°C) demonstrated that the domain was structured, containing 28 ± 4% alpha helical and 25 ± 2% beta sheet content (Figure 5A). Changes in signal intensity at 222 nm were monitored during thermal denaturation. A representative denaturation profile is shown in Figure 5B. The T_m _values from five separate experiments ranged between 55°C to 62°C yielding an average T_m _value of 59 ± 3.5°C. The changes in CD spectra observed during thermal denaturation of CND were not reversible under the conditions used here. To determine the impact of zinc on the secondary structure of CND, apoCND was also monitored using CD spectroscopy under conditions identical to those used for the zinc-bound domain. The CD spectrum of apoCND at 20°C showed a significant loss of signal at 222 nm indicating a decrease in alpha helical content (Figure 5A). The CD spectra of apoCND remained unchanged after titration and incubation with zinc (data not shown), consistent with our finding that apoCND did not rebind zinc after dialysis into zinc-containing buffer (see above). Significantly, the changes in CD spectra observed for apoCND closely resembled those observed for thermally denatured CND (Figure 5A), indicating that removal of zinc ions disrupted folding of the CND to a similar extent as heating to 90°C.

### Cellular localization and mobility of RAG1 non-core CND and bZDD domains

Previously, immunofluorescence studies showed the cellular localization pattern of full length RAG1, as well as various N-terminal non-core fragments [31]. The non-core fragments localized to varying degrees to the overall nucleus, as well as to nuclear subcompartments, such as nucleoli and nuclear speckles. Although the previous study used non-core RAG1 fragments [31], the fragments did not precisely coincide with the structural non-core domains identified in the present study.

To analyze cellular localization properties of different RAG1 regions, we generated GFP fusion constructs of the CND, bZDD, and a combination of both domains (CND+bZDD). In addition, constructs of GFP fused to both full length and core RAG1 were produced. Each GFP fusion construct was transfected into HeLa cells and cellular localization of the expressed protein in fixed cells was visualized by fluorescence microscopy.

**Figure 6:**
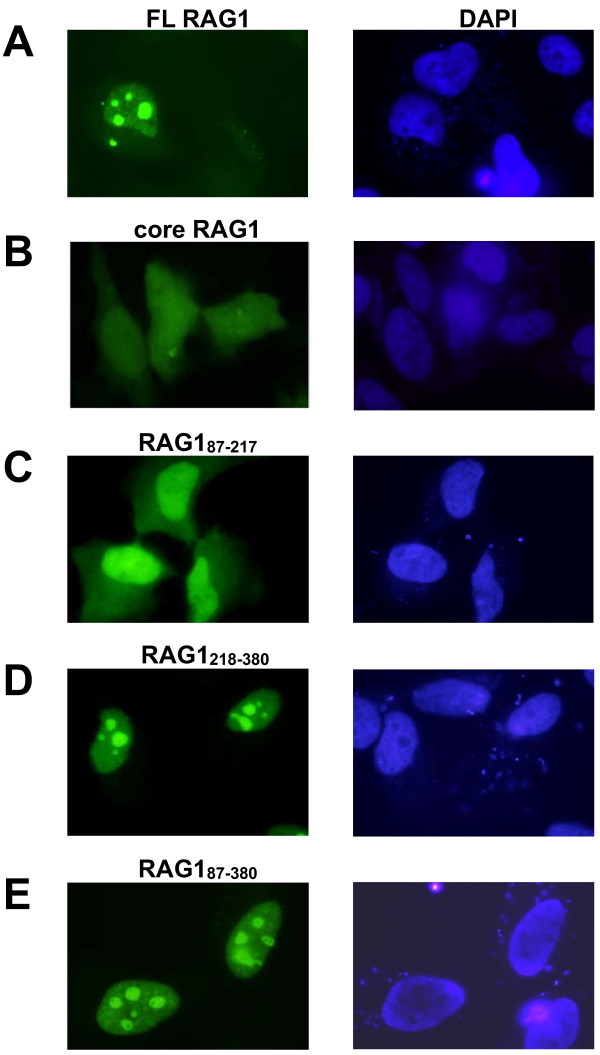
**Nuclear localization of GFP-tagged non-core RAG1 domains**. HeLa cells were transfected with GFP fused to the N-terminus of the indicated RAG1 polypeptides. Following 24-48 hrs cell growth, cells were fixed and stained with DAPI. Left panels: fluorescence microscopy images of HeLa cells expressing **(A) **GFP-full length RAG1, **(B) **GFP-core RAG1, **(C) **GFP-CND (residues 87-217), **(D) **GFP-bZDD (residues 218-380), and **(E) **GFP-(CND+bZDD) (residues 87-380). Right panels: The corresponding nuclei of cells in the left panels stained with DAPI.

GFP-full length RAG1 was localized primarily to nucleoli, consistent with previous results (Figure 6A) [31,66,67]. In contrast, the localization pattern of GFP-core RAG1 was somewhat more variable, with the majority of cells (>70%) containing approximately half (at 46 ± 6%) of the GFP fluorescence signal diffusely localized in the nucleus (see for example leftmost cell in Figure 6B). In the remaining GFP-core RAG1 expressing cells, the protein was more uniformly distributed in both the nucleus and cytoplasm (see for example the middle cell in Figure 6B). Lastly, there is a slight but apparent enrichment of GFP-core RAG1 in nucleoli that is observed in some cells, with bright nuclear spots evident above the fluorescence intensity of protein diffusely distributed throughout the cell (see for example rightmost cell in Figure 6B).

In cells expressing GFP-CND, the localization pattern was similar in all cells analyzed with the majority of the protein localized to the nucleus in each cell (65 ± 6% of the GFP fluorescence signal in the nucleus and the remainder in the cytoplasm) (Figure 6C). The GFP-bZDD fusion protein was predominantly localized to nucleoli, with no protein evident in the cytoplasm (Figure 6D). The combined non-core domains fused to GFP (GFP-CND+bZDD) also localized primarily to nucleoli (Figure 6E). These data show that while the CND could help direct nuclear localization of full length RAG1, the strong nucleolar localization of the full length protein is likely due to the basic region of the bZDD.

**Figure 7:**
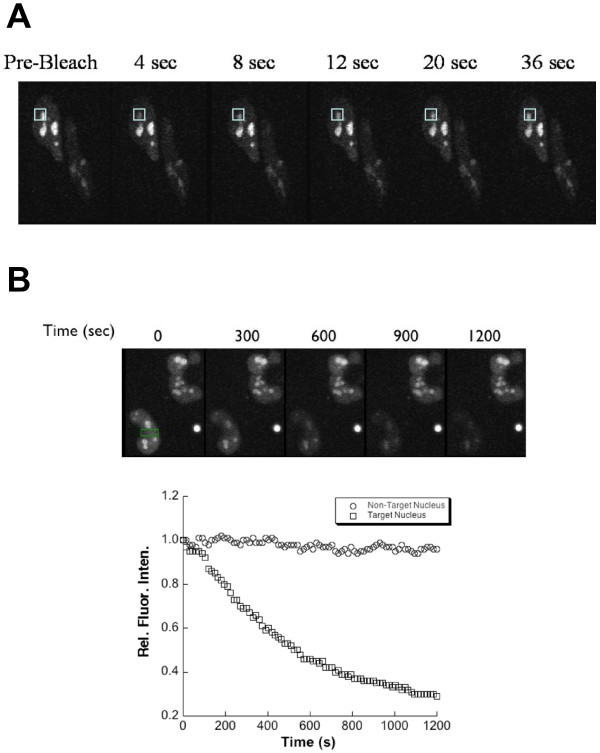
**Fluorescence photobleaching experiments of HeLa cells expressing GFP tagged to a non-core RAG1 region**. **(A) **FRAP measurement of a HeLa cell expressing GFP-(CND+bZDD). The pre-bleach image was collected immediately before photobleaching the indicated region of the nucleus (box). Images were collected at the indicated times following photobleaching. Quantitative analysis of fluorescence intensity in the boxed region showed that the fluorescence signal recovered to 80% of its pre-bleached intensity by 12 s. **(B) **FLIP measurements of HeLa cells expressing GFP-(CND+bZDD). The fluorescent images of two separate cells expressing the GFP fusion protein are shown. The FLIP experiment was performed by repeatedly photobleaching the region indicated by the green rectangle (shown in leftmost image) as described in Methods. An image was collected following each bleach pulse (selected images from 300 to 1200 s after the initial pulse are shown). Plot: The loss of fluorescence intensity of the entire nucleus was measured in each frame and plotted versus frame number. As a control, the fluorescence intensity of the nucleus of the adjacent cell that was not photobleached was measured in parallel.

The relative mobilities of GFP fused to full length and non-core RAG1 domains in live HeLa cells were evaluated using fluorescence photobleaching experiments (Figure 7). In fluorescence recovery after photobleaching (FRAP) experiments, a small region of the cell was photobleached with a brief laser pulse. We monitored fluorescence recovery within the photobleached region over time by collecting a frame every 4 sec following application of the laser pulse. For each GFP fusion protein, fluorescence recovery was essentially complete within approximately 10 sec regardless of whether the protein was diffusely localized in the nucleus (GFP-CND) or predominantly in the nucleoli (GFP-full length RAG1, GFP-bZDD and GFP-(CND+bZDD)). A representative FRAP experiment using a HeLa cell expressing GFP-(CND+bZDD) is shown in Figure 7A. In this example 80% of the fluorescence recovered at 12 sec after photobleaching.

To determine the extent that nucleoli-localized GFP fusion proteins could diffuse from these nuclear subcompartments, fluorescence loss induced by photobleaching (FLIP) experiments were performed with HeLa cells expressing GFP-(CND+bZDD) (Figure 7B). The cell nuclei were repeatedly photobleached with 5 s pulses, with images acquired between pulses. Using this method, the diffusion of fluorophore from all nuclear regions into the path of the laser beam was determined. The loss of GFP fluorescence signals occurred throughout the entire nucleus within the timeframe of the experiment (Figure 7B), indicating that the fusion protein could readily exchange between nucleoli and nucleoplasm subcompartments. Together these FRAP and FLIP results indicate diffusion of the entire population of labeled RAG1 construct, with a mobility comparable to that of other nuclear proteins [68,69].

### Assessing sequence-specific and structure-specific DNA interactions by non-core RAG1 domains

**Figure 8:**
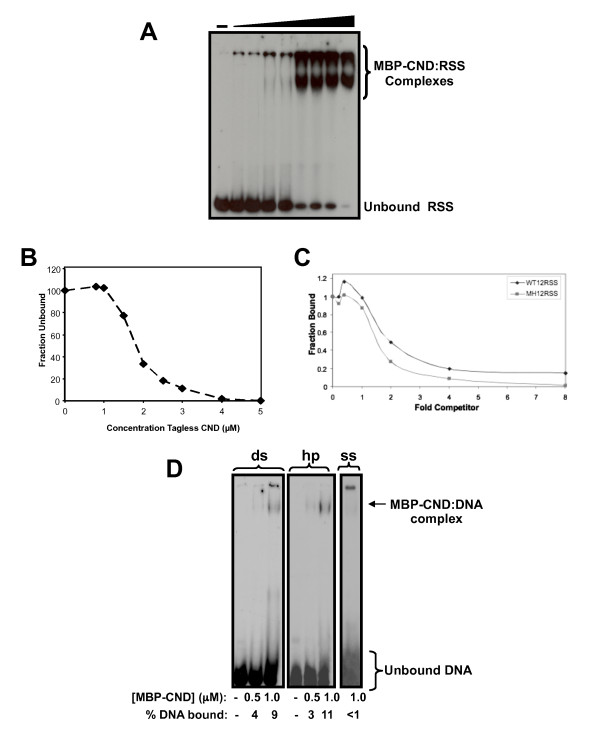
**DNA binding properties of a non-core RAG1 domain**. **(A) **Increasing concentrations ranging from 0-3.0 μM of MBP-CND were titrated into radiolabeled ds DNA substrate containing the RSS. **(B) **Increasing concentrations, ranging from 0-5.0 μM, of tagless CND were titrated into radiolabeled ds WT 12-RSS. Due to difficulty in resolving the protein:DNA complex, the amount of unbound radiolabeled WT 12-RSS was quantified for each data point. Data presented is representative of three independent experiments. **(C) **Increasing concentrations of either WT 12-RSS or mutant nonamer (MN) 12-RSS competed with radiolabeled WT 12-RSS for binding to MBP-CND. For quantification of the competition experiments, the amount of radiolabeled WT 12-RSS bound to CND in the absence of competitor was set at 1.0 and subsequent data are represented as a function of this initial value. Data presented is representative of at least three separate experiments. **(D) **EMSA analysis to compare complex formations between MBP-CND and three separate radiolabeled DNA substrates, which each consist of the sequence corresponding to the 16 nt coding flank of the WT 12-RSS substrate (see Methods). Lanes 1-3 contain the ds 16 bp coding flank, lanes 4-6 contain the coding flank as a fully complementary 32 nt hp substrate, and lane 7 contains the ss 16 nt top strand coding flank sequence. The concentrations of MBP-CND and the percentage of DNA substrate bound are shown below each lane. In panels (A) and (D), protein-DNA complexes were resolved on a 3.5/8% discontinuous, non-denaturing gel.

Although non-core RAG1 is not essential for DNA cleavage and binding activity, it may enhance sequence-specific interactions with the RSS. We tested the ability of non-core RAG1 domains to interact with the RSS using electrophoretic mobility shift assays (EMSA). Increasing concentrations of the isolated domains (fused to MBP) were incubated with a radiolabeled 12-RSS and the extent of complex formation analyzed by nondenaturing polyacrylamide gel electrophoresis. First, we determined that the CND bound to a ds DNA substrate containing the wild type (WT) 12-RSS (Figure 8A). The ability of CND to bind this DNA substrate (tagged with the Oregon Green fluorophore) was confirmed using fluorescence anisotropy experiments (data not shown). The two independent means of analyzing protein:DNA interactions yielded a dissociation constant (K_d_) in the range of 0.8-3.0 μM. Furthermore, tagless CND demonstrated a binding affinity for the DNA substrate comparable to that observed for the MBP-fusion construct (Figure 8B), confirming that the MBP portion of the fusion construct did not contribute or interfere with CND:DNA complex formation. Second, EMSA results showed that the bZDD bound with relatively low affinity (~1-3 μM) to the WT 12-RSS substrate (not shown). Although the isolated ZDD does not associate with DNA [46], the basic region between residues 218-264 (N-terminal to the ZDD) gives the entire bZDD fragment a positive charge (with a predicted pI value of 8.6), which appears to facilitate DNA binding, as previously suggested [31,32,70]. Lastly, MBP-RAG1(1-86) only showed negligible interactions with DNA (not shown).

Even though both the CND and bZDD could bind to the RSS substrate, neither domain could form sequence-specific interactions with the RSS. EMSA assays using nonlabeled WT or mutant RSS substrates as competitors demonstrated that the CND did not show significant specificity for the RSS nonamer (Figure 8C) or RSS heptamer (not shown) of the RSS. Similarly, the bZDD:RSS interaction was not specific for either element of the RSS as judged by competition assays (not shown).

The observed dissociation constants of the isolated CND and bZDD domains for the RSS are only of moderate affinity. If either domain recognized an as yet unidentified DNA sequence, it is likely the binding affinities would be measurably higher to this DNA sequence. To determine this possibility, a systematic evolution of ligands by exponential enrichment (SELEX) method was used [71]. In this method, non-core RAG1 proteins (MBP fused to either the CND alone or to CND+bZDD) were bound to an oligonucleotide duplex containing an internal 25 base pairs (bp) of randomized sequence. The protein-DNA complexes were immobilized on amylose resin, washed extensively, the bound DNA eluted with high salt and temperature, and subsequently amplified by PCR. However, multiple rounds of binding, elution, and amplification failed to increase the binding affinity of either fusion protein to the amplified DNA, indicating that a sequence-specific interaction was not detectable under the conditions used here (not shown).

Recent evidence suggests that full-length versus core RAG1 can more effectively retain the coding end hairpin (hp) in the post-cleavage paired complex containing both 12- and 23-RSS signal ends [72]. It is possible that in the context of the full-length protein, the non-core domains will be juxtaposed close to the coding flanks. Thus, next we asked if the CND or CND+bZDD showed structural specificity for different DNA substrates, including ds, single stranded (ss), or fully complementary hp structures (resembling the coding ends). EMSA analysis revealed that both MBP-CND (Figure 8D) and MBP-CND+bZDD (not shown) bound to the ds and hp substrates with similar affinity, and to ss DNA with significantly lower affinity. Lastly, binding of CND to the DNA substrates was zinc-dependent, as DTPA-treated MBP-CND showed significantly weaker complex formation with the DNA substrates (not shown).

## Discussion

**Figure 9:**
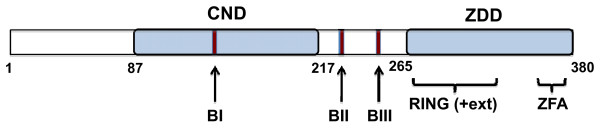
**Features of the N-terminal non-core region of RAG1**. The CND and ZDD structural domains are shown as blue boxes. The RING finger (residues 267-328) and zinc finger A (ZFA; residues 355-376) within the ZDD [46,47] are indicated by brackets. As described previously [47], the RING finger in RAG1, labeled RING(+ext), contains an N-terminal extension that allows formation of the zinc binuclear cluster. Three motifs consisting of clusters of basic residues are shown as red bars. These basic motifs are referred to as BI, BII, and BIII, and consist of residues 141-146 (BI), residues 222-225 (BII), and 243-249 (BIII) [31].

Though most studies of RAG1 have focused on the truncated, core region, the non-core region represents nearly a third of the full-length protein, is well conserved, and has been shown to influence the efficiency and fidelity of recombination [45]. Nevertheless, its structural topology is poorly defined, in large part due to difficulties in characterizing the poorly soluble full-length protein [18,73,74]. To address this issue, we undertook the strategy where discrete structural domains of proteins are defined by their resistance to proteolysis [55], an approach we previously used to identify two separate structural and functional domains in the core region of RAG1 [20]. In the present study, we identified a previously unknown structural domain (the CND) within non-core RAG1, which is capable of folding autonomously. In combination with the ZDD, these domains represent two-thirds of the N-terminal non-core region of RAG1. Notably, regions within the RAG1 gene encoding for residues at/near the CND boundaries do not contain codons rarely found in *E. coli *(not shown). Thus, the formation of the C-terminally truncated MBP-RAG1(1-380) and MBP-RAG1(87-380) fusion proteins during protein purification (see Figures 1 &2) were not due to premature translational termination, but are instead likely due to increased proteolytic susceptibility at the domain boundaries by endogeneous *E. coli *proteases during cell disruption and protein purification. Further, the ability of the CND to form a discrete monomeric species, as opposed to an extensively aggregated form, is further evidence that *bona fide *domain boundaries have been identified. Lastly, the bioinformatic analysis is fully consistent with the CND as a conserved, structured domain flanked by less conserved and highly disordered regions. The CND in relation to other known motifs in the N-terminal non-core region of RAG1 is shown schematically in Figure 9.

Significantly, this study expands the zinc-binding capability of full length RAG1. It has been suggested that the non-core region that includes the CND may bind zinc ions [33,60]. However, this had not been shown directly. We show here that the CND coordinates two zinc ions, which are essential for the folding, stability, and DNA binding functions of this domain. Along with the four zinc ions bound in the bZDD, and at least three zinc ions bound in the core region of RAG1 [75], the full length protein coordinates at least nine zinc ions. These zinc ions are coordinated in distinct zinc-binding motifs, including C_2_H_2 _zinc fingers, a RING domain, a binuclear zinc cluster, and as yet undefined zinc motifs in both the CND and core regions. Along with the PHD zinc-coordinating domain in the non-core region of RAG2 [38], the RAG1:RAG2 V(D)J recombinase is dependent on zinc-coordination in nearly every functional role carried out by the enzyme complex during the recombination reaction, including DNA cleavage [75], macromolecular associations [39,76], and regulatory functions [45].

Here, cellular localization and mobility properties of full length versus non-core and core regions of RAG1 were examined. Full length RAG1 (fused to the C-terminal end of GFP) strongly localized to nucleoli, with some protein present in the nucleoplasm, but absent the cytoplasm, consistent with previous reports [31,66,67]. The nucleoli-localized GFP-RAG1 proteins showed relatively fast mobility between the nucleoli and nucleoplasm. Thus, even in the absence of RAG2, full length RAG1 may gain access to antigen receptor loci, as was indicated by chromatin immunoprecipitation assays [77]. Association of full length RAG1 with nucleoli may be mediated by interactions with RNA [31].

Within the library of RAG1 constructs tested, the core region demonstrated the weakest nuclear localization pattern, with nearly equal distribution between the nucleus and cytoplasm in the majority of cells. These results correspond to previous findings with a RAG1 N-terminal deletion, termed Δ(13-330), which diffusely localized throughout the nucleus and cytoplasm [17].

Nucleolar localization was observed with both the bZDD and core regions, with the bZDD showing the strongest ability to localize to nucleoli. The bZDD and core regions each contain clusters of basic residues, which were previously shown to contribute to nucleolar localization [31]. The bZDD contains the basic motif BIII (Figure 9). The core region contains the basic motifs BIV (residues 826-840) and BV (residues 969-973), which may both influence nuclear and nucleolar localization, albeit to weaker extent than BIII [31]. The CND, which contains BI, another basic motif (Figure 9), is predominantly nuclear with no obvious enrichment in nucleoli. This finding differs somewhat from the mutagenesis studies of Spanopoulou *et al. *[31], in which BI was reported to function as a major contributor to nucleolar localization. Even with this latter discrepancy, our results and others show that the predominant nuclear localization signals of full length RAG1 lie in the N-terminal non-core region.

Full length RAG2 appears to alter the localization pattern of full length RAG1. For example, transfection of the RAG constructs into fibroblast cell lines resulted in co-localization of both proteins to nucleoplasm, but spared the nucleoli [31,67]. Interestingly, Spanopoulou *et al. *[31] showed localization of both RAG proteins at the nuclear periphery in primary thymocytes, as visualized by α-RAG1 immunofluorescence experiments. In a fraction of cells, RAG1 also localized within the nuclear interior in a speckled pattern. However, localization to the nuclear periphery has not been consistently observed in all lymphoid cells tested. For example, following induction of RAG1 and RAG2 expression in Abelson-transformed pre-B cells, α-RAG1 immunofluorescence showed diffuse localization of RAG1 throughout the nucleoplasm, rather than at the nuclear periphery [78]. Additional studies will be important to elucidate factors that may influence localization properties of the RAG proteins.

The observation that CND forms complexes with DNA, albeit with moderate affinity, indicates that non-core RAG1 may participate in maintaining pre- and/or post-cleavage complexes with the RSS and coding flanks during V(D)J recombination. Although the CND and CND+bZDD did not show specific recognition of hairpin ends, the non-core RAG1 region may interact with ds regions of the coding end. Such an interaction could function to properly orient the coding ends within the post-cleavage paired complex, which may be necessary in directing the DNA ends to the NHEJ DNA repair pathway. Notably, Ku was recently shown to co-purify with non-core regions of RAG1 under low stringency conditions [53]. Although the Ku:RAG1 interaction may be indirect, it may be critical for the proper processing of DNA ends. Alternatively, the CND+bZDD region of non-core RAG1 may associate with DNA within the 12- and 23-RSS spacer regions or at the 3' ends of the RSS (adjacent to the nonamer), which could help to lock the V(D)J recombinase on the DNA. A recent study has shown that full length RAG1, in comparison to core RAG1, enhances contacts with the RSS spacer regions, as well as influences the structure of the intervening DNA between the 12/23 RSSs, in a paired complex [72]. Given our results, it is feasible that the CND is at least partially responsible for increased contacts of the full length protein with DNA in the paired complex. Further investigations into the contribution of the non-core regions of RAG1 to the assembly of the RAG-DNA complexes formed during V(D)J recombination will be important to elucidate the configuration of the fully intact V(D)J recombinase on the RSSs in the pre- and post-cleavage complexes.

## Conclusions

All together, these results provide a clearer picture of the architecture of the non-core region of RAG1, as well as the ability of the non-core domains to coordinate metal ions, influence nuclear localization, and interact with DNA. How these domains function together, and with RAG2, to coordinate their activities in the catalytic activity and regulation of V(D)J recombination will provide a significant advancement in our understanding of this intriguing enzyme complex.

## Methods

### Molecular Cloning and Bacterial Expression of Non-core RAG1 Domains

RAG1 constructs were generated as previously described [20], with the following modifications. Briefly, non-core regions of RAG1 were PCR amplified from the full-length murine RAG1 gene using primers designed to create a *BamH*I site and a *Sal*I site at the 5' and 3' ends, respectively. Amplified gene products were then inserted into the appropriate sites within the multiple cloning region of the pMAL-c2 vector (New England Biolabs). MBP fusion proteins of RAG1 residues 1-380, 1-86, 87-217, 87-380, and 218-380 were encoded by plasmids pJLA380, pJLA10, pJLA11, pJLA12, and pJLA218 respectively. Conserved cysteine residues within the pJLA11 vector were mutated to alanine using the QuikChange™ Site-Directed Mutagenesis kit (Stratagene).

Recombinant proteins were expressed in *E. coli *BL21 as previously described [20]. Briefly, transfected cells were grown at 37°C until an OD_600 _of approximately 1.0. Expression of recombinant proteins was induced by addition of IPTG to final concentration of 1 μg/mL, and the culture was grown for an additional 12-15 hours at 25°C. Cells were then harvested and resuspended in Tris Purification Buffer (TPB) (20 mM Tris pH 8.0, 10% glycerol, 50 μM ZnCl_2_, and 5 mM βME) supplemented with 0.5 M NaCl and PMSF at a final concentration of 0.5 mg/mL. Following sonication the cell lysate was incubated at 4°C for 3 hours in the presence of 1 unit/mL RNase A (Sigma) and 32 units/mL DNase I (Roche). Each MBP fusion protein was purified by passing cell lysates over amylose resin (New England Biolabs). The column was washed sequentially with TPB plus 1.5 M NaCl, TPB plus 0.5 M NaCl, and TPB plus 0.2 M NaCl. Protein was eluted from the column in TPB plus 0.2 M NaCl with 10 mM maltose. Subsequently, each MBP fusion protein was then concentrated and fractionated by SEC using a Superdex 75 or Superdex 200 column (GE Healthcare). Column buffer (GFB) contained 20 mM Tris pH 8.0, 0.2 M NaCl, 50 μM ZnCl_2_, and 5 mM βME. Fractions containing the fusion construct were pooled, concentrated, and stored at -20°C in 50% glycerol or at -80°C. The fusion proteins were judged to be > 95% pure based on analysis of Coomassie Blue stained SDS-PAGE gels.

To remove the MBP construct from the CND fusion protein (residues 87-217), a PreScission™ Protease (GE Healthcare) site was generated within the pMAL-c2 vector, 5' of the multiple cloning site, using a QuikChange™ Site-Directed Mutagenesis kit (Stratagene). Purified MBP-CND was digested overnight at 4°C in the presence of 5 units/mg of PreScission™ Protease. Complete digestion of the fusion construct was confirmed by SDS-PAGE and tagless CND was purified by fractionation of the digestion reaction over an SP Sepharose column (GE Healthcare) and elution with a 0.1-1.2 M NaCl gradient. Fractions containing the RAG1 domain were pooled, concentrated, dialyzed into GFB and stored at -80°C.

### MALDI-TOF Mass Spectrometry

Purified MBP-RAG1 fusion proteins were dialyzed overnight at 4°C into 20 mM Tris-HCl, pH 8.0, 50 mM NaCl, 5 mM βME, and 50 μM ZnCl_2_. The proteins were combined with an equal volume of sinapinic acid and analyzed by the Voyager Elite MALDI-TOF mass spectrometer (Applied Biosystems, Framingham, MA) at the National Science Foundation Experimental Program to Stimulate Competitive Research (NSF-Epscor) Oklahoma Laser Mass Spectrometry facility.

### MALLS-SEC

The relative molecular mass of the non-core RAG1 domains were determined by SEC coupled with in-line light scattering detectors as described [79]. SEC was performed using Superdex 200 (with a 20 mL column volume) or Superdex 75 (with a 120 mL column volume) columns to analyze the MBP fusion proteins and tagless RAG1 domains, respectively. The proteins were analyzed at a starting concentration ranging from 3-10 μM, unless noted otherwise. GFB was used as the column buffer.

### Zinc Analysis

Zinc binding ratios were determined by ICP-MS (Oxidor Corporation, Plano, TX) and by FAAS on a SpectrAA-5 spectrophotometer (Varian, Inc., Palo Alto, CA) as previously described [75]. For the FAAS analysis, standards of zinc concentrations ranging from 0.5 to 20 μM were generated from an atomic absorption zinc standard solution (Sigma-Aldrich) and used to establish a standard linear calibration curve. The concentration of zinc in each sample was determined by measuring the absorbance at 213.9 nm after vaporization/atomization of the sample in an air/acetylene-fueled flame. The concentration of zinc in the dialysis buffer was also determined and subtracted as background.

For both methods of measuring zinc content, proteins were dialyzed into zinc-free buffer (ZFB) containing 20 mM Tris pH 8.0, 0.2 M NaCl, and 5 mM βME at 4°C for approximately 40-48 hours with a buffer change at 24 hours. Where indicated, the first round of dialysis was supplemented with 10 mM EDTA, pH 8.0, or 5 mM DTPA.

### Circular Dichroism Spectroscopy

CD spectroscopy experiments were performed using a JASCO J715 Spectropolarimeter with a PTC-348WI peltier temperature controller (Jasco, Corp., Tokyo, Japan) in the OUHSC Physical Biochemistry Equipment Core Facility. The spectral parameters used were as follows: 270-195 nm wavelength range, 0.1 cm cuvette pathlength, and 8-10 accumulations per spectrum. For the wavelength scan analyses, spectra were acquired at 20°C. Protein samples were dialyzed into CD buffer (10 mM Tris-HCl pH 8.0, 0.1 M NaCl, and 1 mM βME). In some cases, the dialysis buffer was supplemented with 10 mM EDTA pH 8.0 or 5 mM DTPA. The protein samples were dialyzed for 48 hours at 4°C in the CD buffer with one to two buffer changes prior to acquiring the CD spectra. Chelating agents were excluded from the final buffer. Protein secondary structural content was predicted using the CDPro software package as previously described [65,80]. Data presented represent the average of results generated by the CDSSTR and CONTINLL programs using three soluble protein reference sets, SP29, SP37, and SP43.

Thermal denaturation studies of CND were performed in CD buffer (listed above). The temperature was raised from 20°C to 90°C at a rate of 30°C/hour with constant monitoring of the intensity of the signal at 222 nm. With each 10°C increase in temperature, the sample was held at a constant temperature for 5 minutes before wavelength scans were taken. The spectral parameters used for the scans during thermal denaturation included: 270-195 nm wavelength range, 0.1 cm cuvette pathlength, and 4 accumulations per spectrum. The fraction folded (f_n_) for each data point was calculated as follows: f_n _= (S^§^-S)/(S^§^-S°) where S is the signal intensity at each intermediate temperature, S° is the signal intensity for the fully folded species and S^§ ^is the signal intensity for the fully denatured species. The T_m_, defined as the point at which f_n _= 0.5, was determined and averaged for five independent denaturation profiles.

### DNA substrates for EMSA

Oligonucleotide substrates were commercially synthesized and PAGE purified (Integrated DNA Technology). The sequence of the top strand of the WT 12-RSS is: d(GATATGGCTCGTCTTACACAGTGATATAGACCTTAACAAAAACCTCCAATCGAGCGGAG) in which the conserved heptamer and nonamer elements are underlined. Mutant 12-RSS substrates were identical to the WT 12-RSS sequence except with the heptamer mutated from CACAGTG to GAGAAGC in the mutant heptamer 12-RSS or the nonamer mutated from ACAAAAACC to AGGCTCTGA in the mutant nonamer 12-RSS. Each top strand WT or mutant 12-RSS was annealed to its respective complement by heating to 95°C for 1 min followed by slow cooling to room temperature.

The sequence of the 16-nt ss DNA substrate is d(GATATGGCTCGTCTTA). The 16 bp ds DNA substrate was formed by annealing the ss coding flank to its complement as described above. The sequence of the 32-nt hp substrate is d(GATATGGCTCGTCTTATAAGACGAGCC ATATC). To form the hp substrate, a low concentration (5 nM) of the oligonucleotide was denatured at 95°C and subsequently quick cooled on ice. Formation of the annealed intramolecular hp substrate, as opposed to an intermolecular duplex form, was confirmed by PAGE prior to each protein:DNA binding experiment.

### EMSA

Each DNA substrate used in EMSA experiments was radiolabeled at the 5' end using [γ-^32^P]ATP and T4 polynucleotide kinase. The top strands of the WT 12-RSS and the 16 bp ds DNA substrates were radiolabeled at the 5' end, and subsequently annealed to their respective complement. The indicated RAG1 domain was incubated with 1 nM ^32^P-labeled DNA substrate at 25°C. The binding buffer (Buffer A) contained 10 mM Tris, pH 8.0, 5 mM MgCl_2_, 2 mM dithiothreitol, 6% glycerol, and 100 mM NaCl. Reactions were resolved on a discontinuous 3.5%/8% nondenaturing polyacrylamide gel as described [65], and analyzed using ImageJ software (National Institutes of Health).

Competition assays were performed under the conditions described above. In these assays, purified MBP-CND was incubated in the presence of 1 nM^32^ P-labeled WT 12-RSS and 0-500 nM of either unlabeled WT 12-RSS or one of the three mutant 12-RSS substrates described above. Each reaction contained 1.5 μM MBP-CND.

### Construction of Green Fluorescent Protein Constructs and Protein Expression in HeLa cells

Constructs encoding GFP-fused to different regions of RAG1 were produced, which yielded expression of GFP-core RAG1 (RAG1 residues 384-1008), GFP-CND (RAG1 residues 87-217), GFP-bZDD (RAG1 residues 218-380), and GFP-CND+bZDD (RAG1 residues 87-380). To produce these constructs, PCR products from the corresponding regions of the RAG1 gene, engineered with SmaI sites at both the 5' and 3' ends of the gene fragment, were sub-cloned 3' to the GFP gene in the mammalian expression vector pWAY5 [81]. GFP-full length RAG1 (RAG1 residues 1-1040) was generated by subcloning the full length RAG1 gene PCR product in frame with GFP into pAcGFP-C1 (Clontech).

HeLa cells were grown on cover slips in six-well plates in Dulbecco's modified Eagle's medium (DMEM) supplemented with antibiotics and 10% fetal bovine serum. Each plasmid construct was transfected into cells using FuGene6 (Roche). Cells transfected with GFP-CND and GFP-bZDD were washed by PBS at 48 hours following transfection; whereas cells transfected with GFP-CND+bZDD were washed by PBS at 24 hours. Subsequently, the cells were fixed in 2% PFA for 20 minutes at room temperature. After three washes in PBS, cover-slips were mounted in UltraCruz mounting medium containing 4',6-diamidino-2-phenylindole (DAPI) (Santa Cruz Biotech).

### Fluorescence Microscopy

Fluorescence cell imaging was performed using a Zeiss LSM-510 META Laser Scanning Confocal Microscopy (Oklahoma Medical Research Foundation Cell Imaging Core Facility). GFP was excited at 488 nm, and emission wavelengths between 530 and 560 nm were collected for imaging. FRAP and FLIP measurements were performed as previously described [69,82]. In brief, for FRAP measurements, a region of nucleus (as indicated) was photobleached using a 29 s pulse of laser illumination. Recovery of fluorescence signal within the bleached regions was monitored by collecting a frame every 4 s. In FLIP experiments, a single spot of the nucleus (as indicated) was repeatedly photobleached with 5 s pulses of laser illumination. Each pulse was followed by image acquisition between consecutive pulses. The same region was photobleached in each pulse, and photobleaching and image acquisition repeated until detectable fluorescence signal was extinguished. Image processing and quantification were performed using iVision (BioVision Technologies, Exton, PA). Decay constants for GFP fluorescence signals were analyzed using nonlinear curve regressions to a single exponential decay.

### Bioinformatic Analysis

Phylogenetic Analysis: The Intrepid (http://phylogenomics.berkeley.edu/INTREPID/index.html) and Conseq (http://consurf.tau.ac.il/) servers were used to identify structurally and/or functionally important residues in the murine RAG1 protein sequence (either non-core only or full length sequence). Both methods collect and align homologous sequences of an input sequence, derive an evolutionary tree, and identify structurally and functionally important positions based on phylogenetic relations between sequence homologs [56,57].

Protein Disorder: The Drippred (http://www.sbc.su.se/~maccallr/disorder/) and Disopred (http://bioinf.cs.ucl.ac.uk/index.php?id=806) servers were used to predict structurally disordered regions in murine non-core RAG1 or full length RAG1. The methods predict regions of disorder by searching for sequence patterns that are not typically found in the protein databank or by sequences that appear in the protein databank sequence records but with coordinates missing from the electron density map ([83] and http://www.sbc.su.se/~maccallr/disorder/).

## List of abbreviations

RSS: recombination signal sequence; WT: wild type; MH: mutant heptamer; MN: mutant nonamer; NBD: nonamer binding domain; CND: central non-core domain; bZDD: basic zinc dimerization domain; MBP: maltose-binding protein; GFP: green fluorescence protein; MALLS-SEC: multi-angle laser light scattering coupled with size-exclusion chromatography; FAAS: flame atomic absorption spectroscopy; ICP-MS: inductively coupled plasma-mass spectrometry; SEC: size-exclusion chromatography; EMSA: electrophoretic mobility shift assay; CD: circular dichroism; FRAP: fluorescence recovery after photobleaching; FLIP: fluorescence loss induced by photobleaching; DAPI: 4',6-diamidino-2-phenylindole; DTPA: diethylenetriamine pentaacetic acid.

## Authors' contributions

Project planning and manuscript composition were performed by JLA and KKR. JLA performed the protein purification, SEC, and MALDI-TOF mass spectrometry experiments in Figure 2; the MALLS-SEC experiments in Figure 4; the circular dichroism spectroscopy experiments in Figure 5; and the EMSA experiments in Figure 8A-C. KKR oversaw data analysis for all experiments, and performed the bioinformatic analysis shown in Figure 3. NSR performed the EMSA experiments in Figure 8D and the SELEX experiments. SZ performed the experiments in Figures 6 and 7 with the assistance of WR. All authors read and approved the final manuscript.
